# Anatomic, isolated complete caudate lobectomy using an anterior transhepatic approach with glissonian pedicle approach and indocyanine green fluorescence guidance: a case report

**DOI:** 10.1093/jscr/rjae505

**Published:** 2024-08-30

**Authors:** Yutaka Endo, Yuta Abe, Minoru Kitago, Yasushi Hasegawa, Shutaro Hori, Masayuki Tanaka, Yutaka Nakano, Motohide Shimazu, Yuko Kitagawa

**Affiliations:** Department of Surgery, Keio University School of Medicine, Shinanomachi 35, Shinjuku, Tokyo 160-8582, Japan; Department of Surgery, Tamakyuryo Hospital, Shimo-oyamadacho 1401, Machida, Tokyo 194-0202, Japan; Department of Surgery, Keio University School of Medicine, Shinanomachi 35, Shinjuku, Tokyo 160-8582, Japan; Department of Surgery, Keio University School of Medicine, Shinanomachi 35, Shinjuku, Tokyo 160-8582, Japan; Department of Surgery, Keio University School of Medicine, Shinanomachi 35, Shinjuku, Tokyo 160-8582, Japan; Department of Surgery, Keio University School of Medicine, Shinanomachi 35, Shinjuku, Tokyo 160-8582, Japan; Department of Surgery, Keio University School of Medicine, Shinanomachi 35, Shinjuku, Tokyo 160-8582, Japan; Department of Surgery, Keio University School of Medicine, Shinanomachi 35, Shinjuku, Tokyo 160-8582, Japan; Department of Surgery, Tamakyuryo Hospital, Shimo-oyamadacho 1401, Machida, Tokyo 194-0202, Japan; Department of Surgery, Keio University School of Medicine, Shinanomachi 35, Shinjuku, Tokyo 160-8582, Japan

**Keywords:** caudate lobectomy, hepatocellular carcinoma, anatomical resection, case report

## Abstract

Hepatocellular carcinoma (HCC) in the caudate lobe presents surgical challenges due to the lack of distinct anatomical landmarks. This case report introduces a novel surgical approach combining Takasaki’s classification and indocyanine green negative counterstaining for precise anatomical caudate lobectomy. A 78-year-old patient with hepatocellular carcinoma in the caudate lobe underwent surgery following preoperative volumetric assessment. The method involved a glissonian approach for both left and right pedicles, coupled with meticulous dissection of hepatic pedicles of the caudate lobe guided by taping of left and right glissonian pedicles, followed by indocyanine green administration for improved visualization of caudate lobe boundaries. The procedure enabled complete tumor resection with minimal blood loss. At 50 months postsurgery, the patient maintains favorable liver function and performance status. This innovative approach offers a promising solution for precise resection of caudate lobe hepatocellular carcinoma, potentially improving surgical outcomes and long-term prognosis.

## Introduction

To completely resect hepatocellular carcinomas (HCCs) in the caudate lobe, isolated caudate lobectomy is a plausible surgical maneuver. However, this procedure has been challenging because no definite landmarks for the boundaries can be observed on the liver surface [1–6]. ‘Takasaki’s classification’ of the liver defines the secondary glissonian pedicles as three branches at the hepatic hilum, and the ‘caudate area’ is defined as the additional area supplied directly from the primary branch [7]. We report a genuine surgical technique for an anatomical caudate lobectomy using Takasaki’s method and ICG negative counterstaining to ensure the complete resection of the tumor’s portal inflow [8, 9].

## Case report

**Figure 1:**
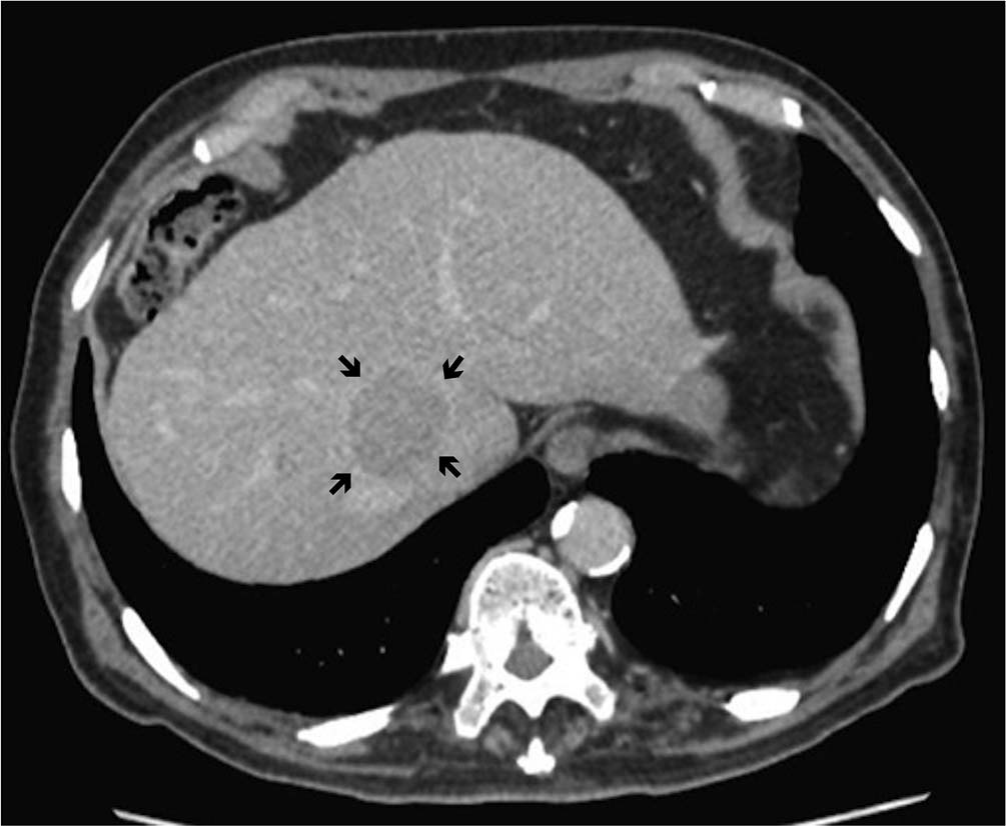
Dynamic computed tomography of the liver. Portal phase of dynamic CT shows a low-density area abutting the RHV, indicating hepatocellular carcinoma located in the caudate lobe (arrows).

A 78-year-old man with a history of hepatitis C was referred to Keio University Hospital for a 4-cm HCC located between the roots of the middle hepatic vein (MHV) and right hepatic vein (RHV) (Fig. 1). The preoperative liver function test was nearly normal (Child-Pugh classification A, ICG retention rate 9.4%). Preoperative volumetry showed that caudate lobectomy with major hepatectomy exceeded the resection limits of the institution (functional remnant volume: 327 ml [31.6%]). His preoperative alpha-feto protein and carbohydrate 19–9 levels were 3.0 ng/ml and 18 U/ml, respectively.

**Figure 2:**
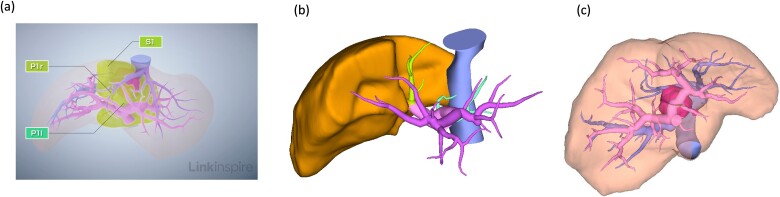
Preoperative radiological assessment of the liver. (a) Three-dimensional reconstruction of preoperative liver anatomy: yellow areas indicate the caudate lobe of the liver (S1) and the pink lesion indicates hepatocellular carcinoma. (b) The glissonian pedicles of the caudate lobe. (c) Portal and hepatic veins are shown as vessels in pink and blue, respectively. The tumor is located in the paracaval portion, adjacent to the right and MHVs.

Three-dimensional reconstruction imaging of the liver demonstrated the following three surgical cruxes (Fig. 2): the tumor was located mainly at the paracaval portion in the caudate lobe (Fig. 2a); resection of the ventral portion of the RHV flow area was required for complete removal of the tumor, as the rightmost glissonian pedicle of the caudate lobe was branched from near the root of the anterior glissonian pedicle and it flowed into the liver underneath the right diaphragm beyond the plane between MHV and RHV (Fig. 2b and c); and a small lesion nodule located at the Spiegel’s lobe was detected, suspicious of a precancerous lesion.

**Figure 3:**
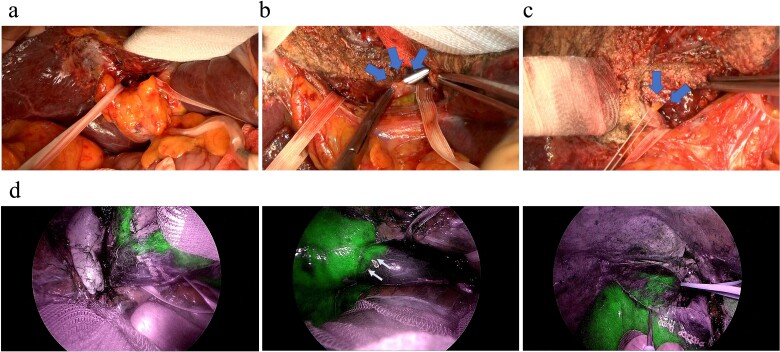
Intraoperative findings during an anatomical complete caudate lobectomy. (a) The left and right glissonian pedicles were secured by a tape. (b) Identification of the Glissonian pedicles of the caudate lobe with a good view after transecting the liver parenchyma, which was achieved using an anterior approach. (c) Ligation of the right-most Glissonian pedicle of the caudate lobe. (d) Definitive delineation of the boundary between the caudate and right lobes by negative counterstaining using indocyanine green fluorescence.

Our method of an anatomical, isolated complete caudate lobectomy was performed as follows (Fig. 3). First, both left and right lobes were fully mobilized, and then, the RHV and the trunk of the left hepatic vein and MHV were isolated. The caudate lobe was mobilized completely from the IVC after dissecting all the short hepatic veins. The left glissonian pedicle was isolated at the left end of the hilar plate (i.e. at the root of the Arantius ligament). Behind the cystic plate, the foot of the right anterior glissonian pedicle was recognized. The glissonian pedicles of the caudate lobe were observed by retracting the taping of the left and right pedicles (Fig. 3a). The liver parenchyma was transected along with the demarcation line by clamping the left glissonian pedicle, the trunk of the MHV, and the Arantius ligament, exposing the ventral side of the caudate lobe. Next, all the glissonian pedicles of the caudate lobe were isolated and ligated from the hilar plate between the left and right glissonian pedicles previously secured with a view from the ventral side because the space between the hilar plate and Laennec capsule was exposed after parenchymal dissection (Fig. 3b). The pedicle that diverged from near the root of the anterior segment glissonian pedicle was carefully identified and dissected (Fig. 3c). After dividing the right-most branch of the glissonian pedicle, ICG was administered systemically. Visualization of the ICG injection was performed using an Olympus laparoscopy system. ICG negative counterstaining provided continuous contrast on the liver surface and visualization of not only the boundary between the caudate lobe and the posterior segment on the dorsal surface of the liver but also the border between the caudate lobe and the anterior segment on the surface of the liver adjacent to the diaphragm (Fig. 3d). The surgical duration was 700 min, and the blood loss volume was 530 ml. The patient experienced a Clavien–Dindo classification II acute kidney injury that was successfully managed with medication and was discharged on postoperative Day 21. Pathological findings revealed that the tumor was well to moderately differentiated with the surgical margin free from tumors with microvascular invasion. During the follow-up period, he experienced a couple of episodes of small intrahepatic recurrences, which were successfully treated through microwave ablation. As of 50 months post-initial surgery, he remains cancer-free with well-preserved liver function and performance status.

The Institutional Review Board approved this case report, and an informed consent was obtained from the patient.

## Discussion

A novel comprehensive approach for an anatomical complete caudate lobectomy described previously was contrived based on Takasaki’s classification and ICG counterstaining in the background of a deeper understanding of the liver anatomy.

The traditional approach for HCC in the caudate lobe was high dorsal resection, reported by Takayama [10]. On the other hand, Kumon’s definition of caudate lobe coincides with Takasaki’s classification of caudate lobe [1]. For the anatomical resection of the caudate lobe, Takasaki’s approach is the most suitable method. Anterior transhepatic approach is performed to provide good visibility and access for glissonian pedicles of the caudate lobe [6]. It is useful, especially when the caudate lobe protrudes beyond the plane between MHV and RHV. The boundaries of the paracaval process differ depending on the patient’s liver anatomy; thus, utilizing ICG negative counterstaining, which makes the border of the caudate lobe be easily recognized, is necessary for the anatomical resection.

Previous studies have demonstrated various surgical options for complete S1 resection, including combined resection (such as left lobe and caudate lobe resection) and high dorsal resection. Compared to combined resection, our method avoids unnecessary extensive resections and helps maintain the sufficient amount of future remnant volume [11]. Furthermore, unlike high dorsal resection, it allows for complete resection along the portal region, particularly beneficial when the tumor extends close to the MHV [12]. However, our method results in more transection surfaces compared to combined resection, making it less simple and requiring detailed anatomical knowledge. Despite its potential, this technique has required further validation, including its application in laparoscopic procedures [13].

## Conclusion

Given that the anatomic characteristics of the caudate lobe are precisely determined based on Takasaki’s classification and ICG counterstaining, this procedure is comprehensive and accessible for the portal inflow of the caudate lobe.

## Supplementary Material

Video_JSCR_rjae505

## Data Availability

The data will be available upon reasonable request to the corresponding author.
